# Integrating Network Toxicology with Molecular Dynamics Simulations to Reveal Key Targets and Binding Mechanisms of Bisphenol A in Pancreatic Ductal Adenocarcinoma

**DOI:** 10.3390/ijms27146439

**Published:** 2026-07-20

**Authors:** Xueru Li, Fan Wu, Zunhan Zhang, Jiayi An, Guoqiang Zhou, Yang Wang, Dandan Zhao, Xiaolu Chen

**Affiliations:** 1 College of Biological Sciences and Technology, Yili Normal University, Yining 835000, China; lixueruc@163.com (X.L.); 241305012@ylnu.edu.cn (Z.Z.); 231002056@ylnu.edu.cn (J.A.); 231002098@ylnu.edu.cn (G.Z.); 18188394528@163.com (Y.W.); 23123@ylnu.edu.cn (D.Z.); 2 Chongqing Key Laboratory of Interface Process and Soil Health, College of Resources and Environment, Southwest University, Chongqing 400716, China; wufanylnu_edu@163.com

**Keywords:** bisphenol A, pancreatic ductal adenocarcinoma, network toxicology, molecular docking, molecular dynamics simulation

## Abstract

Bisphenol A (BPA) is a potential risk factor for pancreatic ductal adenocarcinoma (PDAC). This study integrated network toxicology, molecular docking, molecular dynamics (MD) simulations, and TCGA clinical data analysis to explore the potential molecular mechanisms linking BPA exposure to PDAC risk. BPA–PDAC intersection targets were identified through multi-database screening, followed by protein–protein interaction (PPI) network construction to screen core hub genes. A total of 10 core hub genes were identified via PPI analysis combined with the Maximal Clique Centrality (MCC) algorithm. Molecular docking demonstrated that ESR1 exhibited one of the strongest binding affinities for BPA (−8.2 kcal/mol), and MD simulations confirmed favorable thermodynamic stability of the BPA–ESR1 complex. TCGA analysis revealed stage-dependent expression patterns: early stages showed downregulation of TP53 and BCL2, whereas advanced stages showed upregulation of BCL2L1, HSP90AA1, and HSP90AB1, while ESR1, HIF1A, and PARP1 remained consistently low. These findings suggest that BPA may promote PDAC progression by disrupting ERα-mediated endocrine signaling and impairing DNA repair through PARP1 interference, providing candidate molecular targets and a hypothesis-generating foundation for pancreatic cancer risk assessment, warranting further experimental validation.

## 1. Introduction

Pancreatic cancer is one of the most lethal malignancies worldwide, with a five-year survival rate below 10% [1,2,3]. Pancreatic ductal adenocarcinoma (PDAC) accounts for over 90% of all pancreatic cancer cases and is characterized by high invasiveness, insidious early symptoms, and limited therapeutic options [4,5]. The development and progression of PDAC involve complex interactions among genetic [6,7], environmental [8], and hormonal [9] factors.

Bisphenol A (BPA) is a key monomer used in the production of epoxy resins and polycarbonate plastics and is widely applied in food-contact packaging, medical devices, and consumer electronics. Although polymerization can incorporate BPA into the plastic matrix, trace amounts of the compound may migrate into the contents, particularly under conditions of heating or abrasion [10,11]. Biomonitoring data indicate that BPA is ubiquitously detected in human urine samples [12].

As a typical endocrine-disrupting chemical, BPA exhibits estrogenic activity and can exert biological effects through ERα, ERβ, and GPER (G Protein-Coupled Estrogen Receptor) pathways [13]. In terms of metabolic toxicity, numerous studies have demonstrated that BPA exposure is closely associated with metabolic disorders, including obesity, insulin resistance, and type 2 diabetes [14,15]. BPA can induce pancreatic β-cell apoptosis, disrupt insulin secretion, and exert toxic effects on pancreatic islets through multiple receptor-mediated pathways [13]. Regarding immunoregulation, BPA possesses immunosuppressive activity, can downregulate T-cell function, and can compromise the antioxidant enzyme system, thereby disrupting immune homeostasis [16]. Obesity, a common phenotype of the aforementioned metabolic disorders, has been shown to exacerbate PDAC progression by promoting myeloid cell infiltration and fibrosis in the tumor microenvironment [17]. Chronic inflammation, insulin resistance, and metabolic reprogramming collectively create a permissive microenvironment for PDAC development [18].

Although accumulating evidence suggests a potential association between BPA exposure and pancreatic cancer risk, existing studies have largely focused on isolated pathways or single-cell models [13,19], lacking comprehensive analysis of the BPA–PDAC molecular network from a systems biology perspective, as well as dynamic validation incorporating clinical staging characteristics. Network toxicology, as an emerging approach integrating systems biology, bioinformatics, and computational toxicology, enables the systematic elucidation of complex interaction networks between chemicals and diseases. The present study integrated TCGA stage-specific dynamic validation with MD simulations to systematically explore the potential molecular mechanisms by which BPA exposure may be linked to PDAC, providing novel scientific evidence for the risk assessment of environmental endocrine-disrupting chemicals.

## 2. Results

### 2.1. BPA Toxicity Prediction Analysis

**Figure 1:**
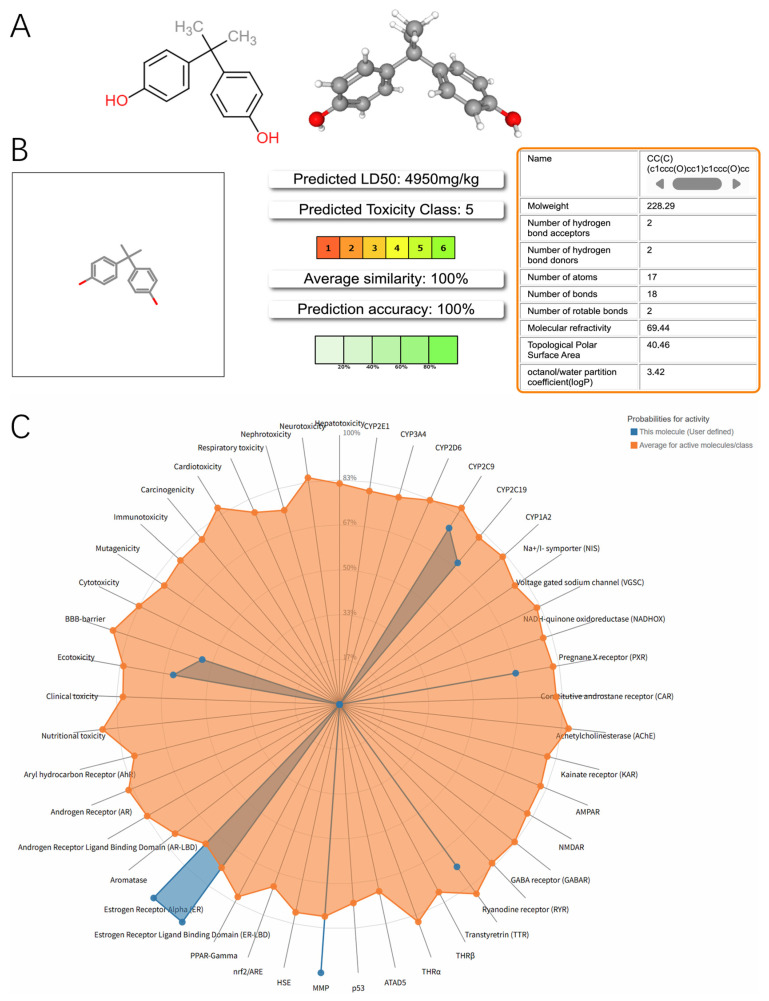
BPA toxicity prediction. (**A**) BPA chemical structure; (**B**) Toxicity analysis; (**C**) Molecular initiating events.

The chemical structure and 3D conformation of BPA are shown in Figure 1A. Toxicity prediction results from ProTox 3.0 and ADMETlab 2.0 indicated that BPA had a predicted LD50 of 4950 mg/kg, classified as toxicity class 5 (relatively low toxicity) (Figure 1B) [20,21]. Its organ toxicity profile included hepatotoxicity, nephrotoxicity, neurotoxicity, respiratory toxicity, and cardiotoxicity. Molecular initiating event analysis suggested that BPA may interact with molecular targets including ERα, aromatase, and the constitutive androstane receptor (CAR), indicating its potential endocrine-disrupting and carcinogenic risks (Figure 1C).

### 2.2. Identification of BPA–PDAC Intersection Targets

**Figure 2:**
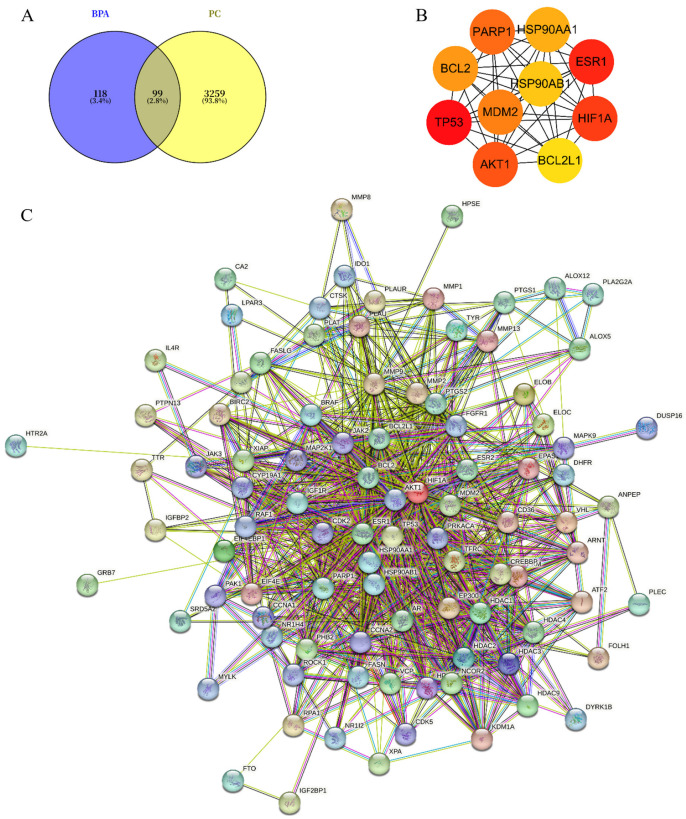
Screening of BPA-related targets in pancreatic cancer. (**A**) Venn diagram of BPA targets and pancreatic cancer-related targets. (**B**) Top 10 core targets ranked by degree value in the PPI network. (**C**) PPI network of intersection targets constructed using the STRING database.

A total of 168 BPA-related targets were obtained from the ChEMBL database, and 106 potential targets were obtained from the SwissTargetPrediction platform. After integration and deduplication, 217 BPA candidate targets were obtained. PDAC-related targets were retrieved from the GeneCards, OMIM, and TTD databases, yielding 2939, 520, and 99 targets, respectively, which were integrated and deduplicated to obtain 3359 PDAC disease targets. Venn diagram analysis of the 217 BPA candidate targets against the PDAC disease targets identified 99 intersection genes (Figure 2A), which were prioritized as candidate targets for BPA-associated PDAC risk for subsequent network and functional analyses.

### 2.3. PPI Network Construction and Core Hub Gene Screening

The 99 intersection targets were imported into the STRING database to construct a PPI network. After removing disconnected nodes, the network contained 95 nodes and 1816 interaction edges, with an average node degree of 19.116 (Figure 2B), indicating high network connectivity. The network data were imported into Cytoscape 3.10.3, and the MCC algorithm of the CytoHubba plugin was used to screen the top 10 core hub genes: TP53, ESR1, HIF1A, AKT1, PARP1, MDM2, BCL2, HSP90AA1, HSP90AB1, and BCL2L1 (Figure 2C). Detailed hub gene frequency data are provided in Supplementary Table S1.

### 2.4. GO Functional and KEGG Pathway Enrichment Analysis

**Figure 3:**
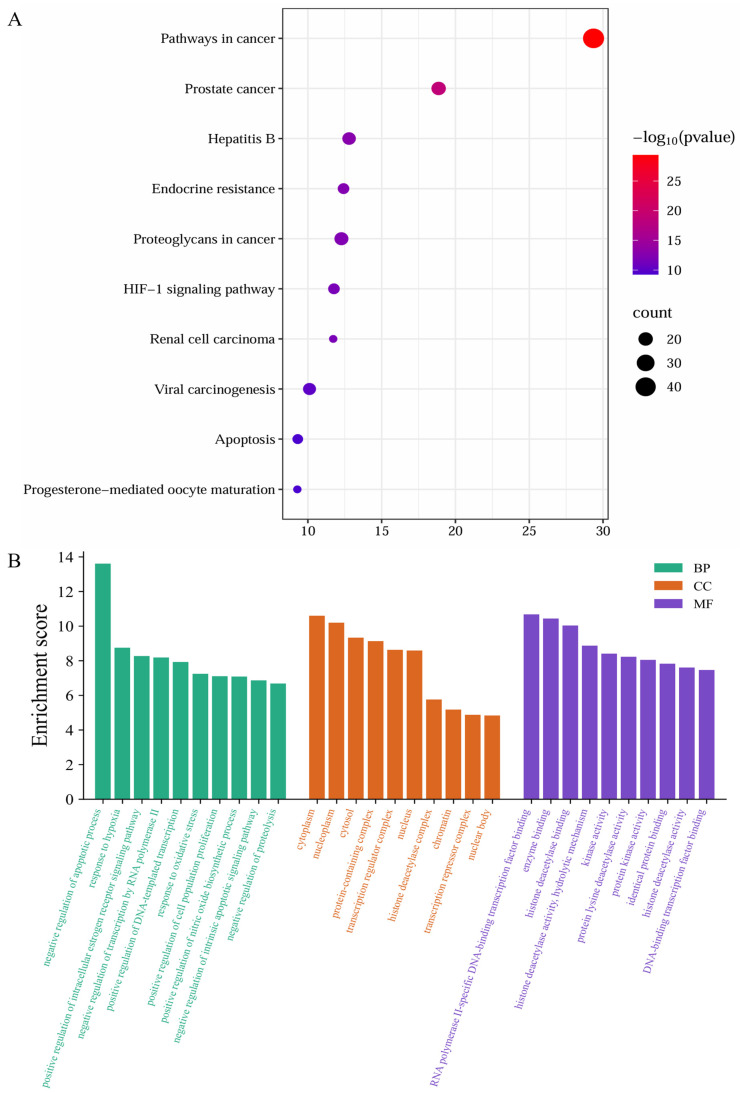
Functional enrichment analysis of core targets. (**A**) Top 10 enriched KEGG pathways. (**B**) GO enrichment analysis of biological process (BP), cellular component (CC), and molecular function (MF).

KEGG pathway enrichment analysis yielded 132 terms (Figure 3A). After sorting in ascending order by FDR, the pathway with the highest confidence was “Pathways in cancer.” GO functional enrichment analysis identified 426 significant terms (*p* < 0.05), including 275 in Biological Process (BP), 57 in Cellular Component (CC), and 94 in Molecular Function (MF) (Figure 3B). The top 10 enriched terms in each category showed that the predominantly enriched BP was “response to oxidative stress,” the CC was “cytoplasm,” and the MF was “RNA polymerase II-specific DNA-binding transcription factor binding”. The complete lists of enriched KEGG pathways (132 terms) and GO terms (426 terms) are provided in Supplementary Table S2.

### 2.5. Molecular Docking Analysis

**Figure 4:**
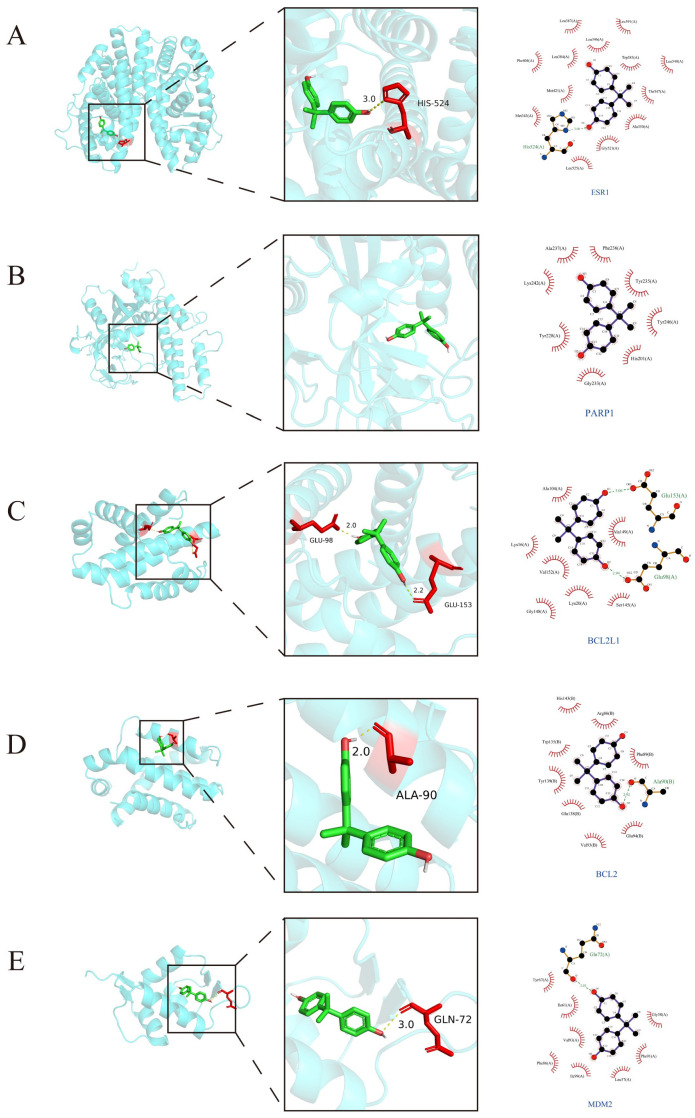
Molecular docking results of bisphenol A (BPA) with core targets. (**A**) BPA–ESR1; (**B**) BPA–PARP1; (**C**) BPA–BCL2L1; (**D**) BPA–BCL2; (**E**) BPA–MDM2. The 3D binding poses and 2D interaction diagrams illustrate the binding modes of BPA with each target protein. Left panel: The green region represents the small molecule (BPA). Yellow dashed lines indicate hydrogen bonds, and the red segments denote amino acid residues that form hydrogen bonds with the small molecule.Right panel: The small molecule is shown at the center. Green lines represent hydrogen bonds, with the other ends of the lines connecting to the corresponding amino acid residues.

To validate the binding activity of BPA with the targets, molecular docking simulations were performed on the core targets, and the five results with the best docking binding energies were visualized (Figure 4). BPA exhibited favorable predicted binding interactions with all 10 core targets. Among them, ESR1 (PDB: 1L2I, −8.2 kcal/mol), PARP1 (−8.1 kcal/mol), and BCL2L1 (−7.2 kcal/mol) showed the strongest predicted interactions. However, the 0.1 kcal/mol difference between ESR1 and PARP1 falls within the typical uncertainty range of molecular docking calculations, suggesting that multiple targets may be equally relevant. Binding mode analysis indicated that BPA primarily interacts with the target proteins through hydrogen bonds and hydrophobic interactions.

### 2.6. Molecular Dynamics Simulation Analysis

Based on the molecular docking results, a 100 ns MD simulation was further conducted on the BPA–ESR1 (1L2I) complex to investigate its interaction mechanism and stability in detail.

**Figure 5:**
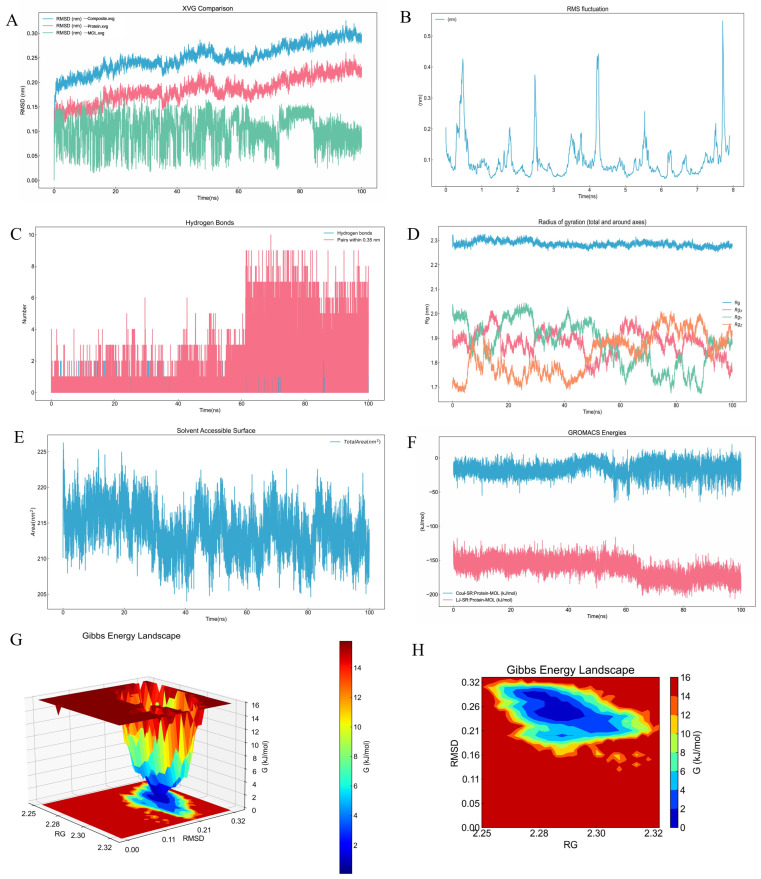
Molecular dynamics simulation analysis of the BPA–ESR1. (**A**) RMSD; (**B**) RMSF; (**C**) Hydrogen bond number; (**D**) Rg; (**E**) SASA; (**F**) Energy; (**G**) 2D Free energy landscape; (**H**) 3D Free energy landscape.

RMSD analysis revealed that, during the 100 ns simulation, the ligand RMSD curve fluctuated predominantly between 0.05 and 0.15 nm. The complex backbone RMSD converged into a single trajectory cluster after 10 ns, demonstrating favorable conformational stability of the protein backbone and ligand, with the system reaching thermodynamic equilibrium (Figure 5A). RMSF analysis showed that the atomic RMSF values were predominantly concentrated between 0.05 and 0.20 nm, confirming the relative stability of atomic flexibility and side-chain motions throughout the simulation (Figure 5B). During the latter half of the simulation (50–100 ns), an average of 4–6 hydrogen bonds were observed between the protein and the small molecule (Figure 5C). Radius of gyration (Rg) analysis indicated an average Rg value of 2.3 nm for the complex, suggesting a relatively open conformation; the Rg rapidly reached equilibrium after 10 ns, demonstrating high structural flexibility and stability (Figure 5D). Solvent-accessible surface area (SASA) analysis showed that the complex SASA values ranged from 210 to 220 nm^2^, consistent with the Rg results, indicating that the complex maintained a certain degree of structural flexibility while preserving stable binding (Figure 5E). Non-bonded interaction energy analysis revealed that the short-range Coulombic interaction energy between BPA and ESR1 was −15.67 kJ/mol, and the short-range Lennard-Jones potential energy was −162.59 kJ/mol (Figure 5F).

The two-dimensional free energy landscape constructed using the PC1–PC2 reaction coordinates presented a clear contour topology. The color scale was uniformly calibrated to Gibbs free energy (0–20 kJ/mol), with deep blue basins (<2 kJ/mol) corresponding to the global minimum conformation and red high-energy regions (>17.5 kJ/mol) marking transition states. The contour density was most concentrated in the range of Rg ∈ [2.25, 2.31] nm and RMSD ∈ [0.17, 0.32] nm, indicating the presence of multiple metastable states in this region (Figure 5G). The three-dimensional free energy surface, generated by height-mapping energy values, formed a distinct funnel-shaped energy landscape. The lowest energy basin was located at Rg = 2.28 nm and RMSD = 0.26 nm (<0 kJ/mol). The 3D view clearly revealed three major conformational subpopulations that corresponded precisely to the contour ring structures in the 2D plot (Figure 5H). These results were mutually corroborated by the single-cluster convergence observed in RMSD analysis and the stable fluctuation of Rg, confirming from a thermodynamic perspective that BPA and ESR1 formed a stable complex with high binding affinity and conformational specificity.

### 2.7. Stage-Specific Expression Profiles of Core Hub Genes in TCGA-PAAD

**Figure 6:**
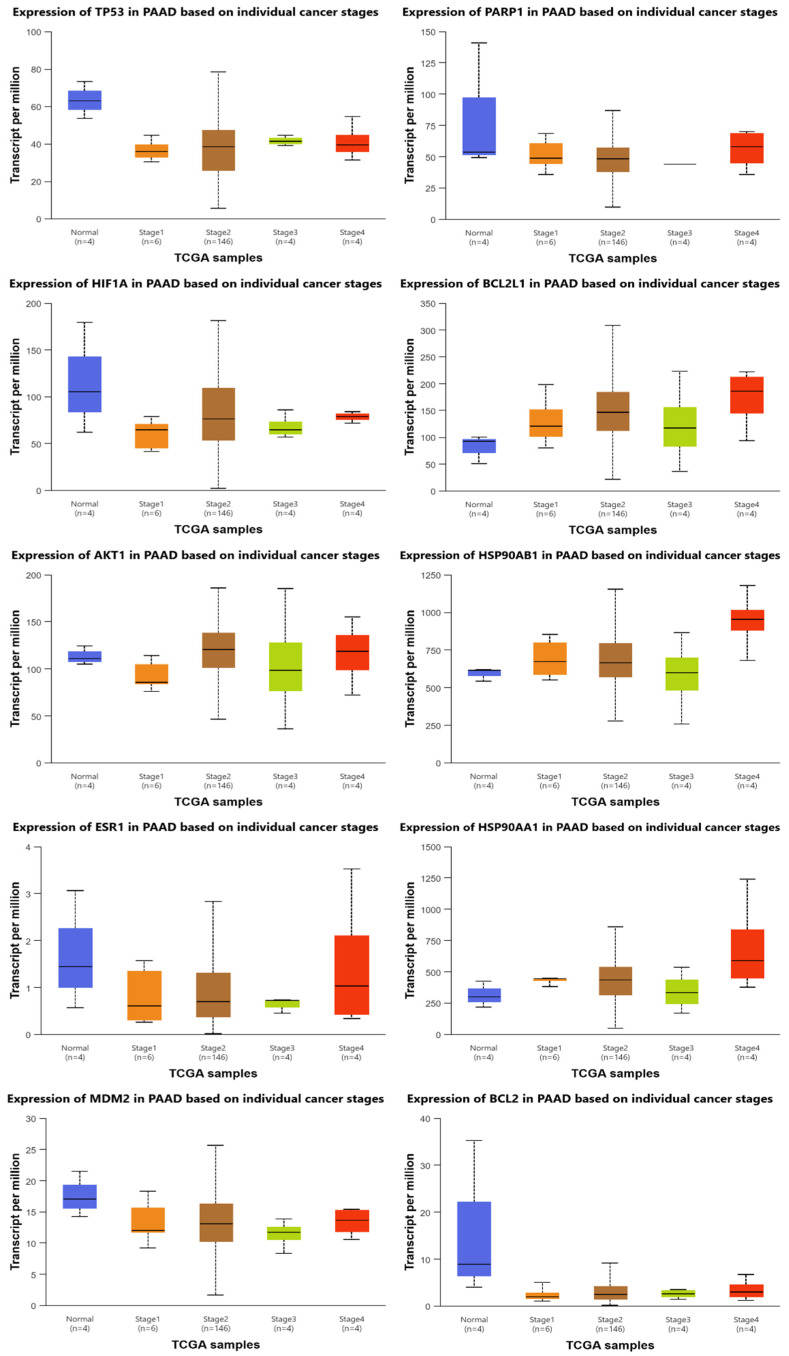
Clinical data analysis of core hub genes.

Stage-specific expression profiling of the 10 core hub genes was performed based on the TCGA-PAAD cohort (Figure 6). The results revealed that these genes exhibited distinct patterns across different clinical stages of PDAC.

Early downregulated genes: TP53 was significantly decreased compared to normal tissue as early as Stage I, suggesting early loss of genomic surveillance function. BCL2 was sharply downregulated in tumor tissues. ESR1, HIF1A, and PARP1 exhibited consistently low expression across all stages, showing substantial downregulation relative to normal tissues, suggesting that transcriptional repression of these genes may constitute an early molecular event in PDAC development.

Late upregulated genes: BCL2L1, HSP90AA1, and HSP90AB1 showed progressive upregulation with advancing stage, reaching peak levels at Stage IV, suggesting that anti-apoptotic signaling and chaperone-mediated proteostasis networks are coordinately activated in advanced tumors.

Genes with fluctuating expression: MDM2 and AKT1 showed no significant monotonic trends across clinical stages, with transient elevation observed at Stage II, suggesting that their oncogenic functions may be regulated primarily through post-translational modifications rather than linear transcriptional changes.

## 3. Discussion

The present study systematically elucidated the molecular mechanisms by which BPA may induce PDAC through multi-target synergistic effects by integrating network toxicology and computational simulation approaches. The 10 identified core hub genes are widely involved in key biological processes including cell proliferation, apoptosis, DNA damage repair, and epigenetic regulation.

It should be emphasized that the TCGA dataset does not contain BPA exposure information; therefore, the observed expression patterns cannot be directly attributed to BPA, but rather serve as complementary descriptive evidence of the potential clinical relevance of the core genes in PDAC progression.

It is important to emphasize that the computational methods employed in this study—including network toxicology, molecular docking, and MD simulations—are predictive and hypothesis-generating tools. While they provide valuable insights into potential molecular interactions and pathways, they do not constitute direct evidence of biological function. The proposed mechanisms, including BPA–ESR1 binding and its downstream effects on endocrine signaling and DNA repair pathways, require experimental verification through surface plasmon resonance or isothermal titration calorimetry for binding affinity validation, as well as functional studies in PDAC cell lines (e.g., proliferation, apoptosis, and DNA damage repair assays following BPA exposure) and in vivo animal models before they can be confirmed as biological facts. Furthermore, all mechanistic interpretations presented here should be viewed as hypotheses to be tested experimentally, rather than demonstrated biological mechanisms.

ESR1 encodes ERα, the core receptor mediating estrogen signaling, and recent studies have demonstrated that ERα is expressed in pancreatic adenocarcinoma and holds prognostic significance [22]. In this study, molecular docking revealed that BPA exhibited favorable binding affinity with ESR1 (−8.2 kcal/mol), and the 100 ns MD simulation confirmed the excellent thermodynamic stability of this complex, with the Rg value stabilizing at 2.3 nm, SASA fluctuations indicating moderate surface flexibility, and FEL analysis further revealing a well-defined global energy minimum conformation. Collectively, these in silico findings provide atomic-level support for ESR1 as a predicted direct interaction target of BPA. TCGA data showed that ESR1 was nearly abolished (downregulated by over 80%) in PDAC tumor tissues compared to normal tissues, suggesting that transcriptional repression of this core hub gene may constitute an early molecular event in PDAC development. Taken together, the favorable binding profile predicted by docking and MD simulations, combined with the marked downregulation observed in clinical samples, supports the hypothesis that ESR1-mediated endocrine signaling disruption may represent a key molecular event in BPA-associated PDAC progression [23]. Pancreatic tissue is not only an exocrine organ but also possesses endocrine function. 17β-Estradiol exerts anti-apoptotic protective effects through ERα, ERβ, and GPER [24], whereas BPA, as an exogenous estrogen mimic, may interfere with the tumor-suppressive function of endogenous estrogen by competitively binding ERα [13].

In addition to ESR1, BPA exhibited favorable binding affinity with PARP1 (−8.1 kcal/mol). PARP1 is a key enzyme in single-strand DNA break repair, and its functional status directly affects genomic stability [25,26]. TCGA data showed that PARP1 was consistently underexpressed in PDAC. Based on docking results, it is postulated that BPA may occupy the catalytic domain or NAD^+^ binding pocket of PARP1, thereby interfering with its enzymatic function and potentially amplifying BPA-induced oxidative stress damage. In pancreatic epithelial cells, sustained DNA damage accompanied by repair pathway inhibition may lead to cumulative genomic instability, providing a mutational basis for PDAC development [27,28].

As the “guardian of the genome,” TP53 plays a central role in cell cycle regulation and apoptosis induction [29,30]. TCGA stage-specific analysis showed that TP53 was significantly downregulated to approximately 30% of normal levels as early as Stage I, indicating that its functional loss is an early critical event in the context of BPA-associated PDAC risk. Although the direct binding energy between BPA and TP53 was relatively weak (−5.8 kcal/mol), TP53 possessed the highest topological importance in the PPI network. As an environmental estrogen, BPA can interfere with signaling pathways mediated by estrogen receptor alpha (encoded by ESR1) [31]. In multiple cancer models, associations between BPA and TP53 functional abnormalities have been observed [32,33]. Existing evidence suggests that BPA may affect processes such as cell cycle regulation, oxidative stress, and tumor microenvironment remodeling through endocrine-disrupting effects [32]. Notably, integrative network toxicology and molecular docking analyses have demonstrated that BPA can stably bind to HSP90AA1 and HSP90AB1, modulating oncogenic and immune-inflammatory pathways in esophageal carcinoma [34]. As molecular chaperones, HSP90 family proteins are essential for maintaining proteostasis and stabilizing immune signaling client proteins within the tumor microenvironment [35,36], suggesting that BPA may disrupt these functions through direct HSP90 interference.

Overexpression of BCL2 family members (including BCL2, BCL-xL, and MCL1) is a universal mechanism by which tumor cells evade mitochondrial apoptosis pathways, among which BCL-xL occupies a prominent position in survival dependence across multiple solid tumors [37,38]. TCGA data showed that BCL2 was sharply downregulated in tumor tissues, whereas BCL2L1 (BCL-xL) exhibited progressive upregulation, reaching peak levels at Stage IV. The sharp downregulation of BCL2 in PDAC is consistent with the intrinsic molecular characteristics of this tumor type. Unlike hematological malignancies (e.g., follicular lymphoma), where BCL2 overexpression drives survival, solid tumors, particularly PDAC, primarily rely on BCL-xL (BCL2L1) and MCL1 to maintain mitochondrial apoptosis resistance, and BCL2 itself is not a critical survival dependency factor in PDAC. The low BCL2 expression observed in TCGA may reflect a switch in the anti-apoptotic network during acinar-to-ductal metaplasia: normal pancreatic acinar cells highly express BCL2 to maintain homeostasis, while clonal selection pressure during tumor progression drives cells toward the more efficient BCL-xL-dependent survival mechanism. The progressive upregulation of BCL2L1 is highly consistent with published mechanistic studies. CRISPR-Cas9 genome-wide screening studies have unequivocally demonstrated that BCL-xL is a key driver of gemcitabine resistance in PDAC cells, and targeted inhibition of BCL2L1 (e.g., using the BCL-xL-specific degrader DT2216) can effectively reverse the resistant phenotype and restore chemosensitivity [39].

Multiple studies have consistently demonstrated that HIF1A is significantly overexpressed in PDAC tissues and drives gemcitabine resistance and poor prognosis by forming a positive feedback loop with HIF1A-AS1 to enhance glycolysis [40,41]. In this study, the expression pattern of HIF1A in PDAC revealed a complex dissociation between transcriptomic observations and functional mechanisms. TCGA-PAAD data showed that HIF1A mRNA was expressed at higher levels in normal pancreatic tissue but decreased in Stage I–IV tumor tissues. Notably, the “normal controls” in TCGA-PAAD are histologically normal adjacent pancreatic tissues rather than healthy pancreatic tissues from non-cancer individuals. Aran et al. [42] demonstrated in their pan-cancer transcriptomic analysis that tumor-adjacent normal tissues (NATs) occupy a unique intermediate state between health and tumor at the molecular level, characterized by enrichment of hypoxia and inflammatory gene signatures, with an influence extending up to 4 cm from the tumor margin. Furthermore, Lee et al. [43] confirmed that HIF1α was widely accumulated in inflamed pancreatic tissues in acute and chronic pancreatitis models, while being barely detectable in normal pancreas, with 70% of human chronic pancreatitis samples showing HIF1α positivity (7/10). Therefore, the elevated baseline HIF1A expression in TCGA adjacent tissues, due to concomitant chronic inflammation and local hypoxic microenvironment, may artifactually amplify the magnitude of “relative downregulation” in tumor tissues. In this study, the coordinated upregulation of HSP90AA1 and HSP90AB1 in advanced stages is consistent with the hypothesis that these chaperones contribute to maintaining HIF1A function. As molecular chaperones, HSP90 family proteins can stabilize the conformation and function of client proteins (including AKT1 and HIF1A) [44], and their significant elevation in advanced stages suggests that BPA may assist in the correct folding of upregulated proteins such as BCL2L1 through HSP90-mediated proteostasis, thereby constructing a proteostasis microenvironment conducive to tumor progression [44].

AKT1 and MDM2 showed minimal expression fluctuations across TCGA stages; however, both possessed high topological importance in the PPI network. AKT1, as a core node of the PI3K/AKT pathway, is primarily regulated by its phosphorylation status [45,46]. MDM2, as the E3 ubiquitin ligase of TP53, may be downregulated as a compensatory feedback response to TP53 loss [47,48]. We hypothesize that BPA may affect the function of these two nodes through post-translational modifications rather than transcriptional regulation, thereby perturbing downstream signaling networks without significantly altering their mRNA levels.

Several limitations should be acknowledged. First, the network toxicology approach relies on computational predictions, and the identified BPA–PDAC targets have not been experimentally validated through in vitro or in vivo assays. Although molecular docking and MD simulations provide theoretical support, these in silico predictions remain hypothetical without confirmatory data (e.g., SPR, ITC) or functional evidence from cellular models (e.g., CRISPR/Cas9-mediated gene knockout, BPA exposure assays in PDAC cell lines) and animal studies. Second, SwissTargetPrediction identifies targets based on 2D structural similarity, which may miss non-classical targets that interact through allosteric or metabolite-mediated mechanisms. Third, the 100 ns MD simulation is sufficient for local stability analysis but may be inadequate for large-scale conformational transitions; longer simulations (≥500 ns) or enhanced sampling would better characterize binding thermodynamics. Fourth, TCGA transcriptomic data reflect mRNA levels only and cannot capture post-translational modifications or protein activity states; validation at the proteomic level (e.g., CPTAC) is warranted. Fifth, a quantitative dose–response relationship between BPA exposure and PDAC risk was not established. Finally, MD simulations were limited to BPA–ESR1; the dynamic binding of other high-affinity targets (PARP1: −8.1, BCL2L1: −7.2 kcal/mol) requires parallel validation. Additionally, the 99 intersection genes were identified through database overlap without statistical enrichment testing (e.g., hypergeometric test or Fisher’s exact test); therefore, this gene set should be interpreted as a computationally prioritized candidate list rather than a statistically validated signature. Despite these limitations, this study provides a systematic framework for understanding the BPA–PDAC molecular link and identifies promising targets for future investigation.

## 4. Materials and Methods

### 4.1. BPA Toxicity Prediction

The online toxicology prediction platforms ProTox 3.0 (https://tox.charite.de) (accessed on 1 February 2026) and ADMETlab 2.0 (https://admetmesh.scbdd.com) (accessed on 1 February 2026) were employed to analyze the toxicity profile of BPA based on its SMILES structure, including organ toxicity (hepatotoxicity, nephrotoxicity, neurotoxicity, etc.) and molecular initiating events.

### 4.2. BPA Potential Target Prediction

The chemical structure and SMILES notation of bisphenol A (BPA) were retrieved from the ChEMBL database (https://www.ebi.ac.uk/chembl/) (accessed on 2 February 2026)). The SMILES string was imported into the SwissTargetPrediction online platform (http://www.swisstargetprediction.ch/) (accessed on 2 February 2026), with the species parameter set to Homo sapiens, for in silico prediction of potential interaction targets. The validated targets from the ChEMBL database and the predicted targets from SwissTargetPrediction were subsequently integrated, and duplicates were eliminated based on UniProt IDs to construct the BPA-related target set.

### 4.3. PDAC-Related Target Collection and Intersection Gene Identification

PDAC-related targets were retrieved from the GeneCards database (https://www.genecards.org/ (accessed on 2 February 2026), relevance score ≥ 10.0), OMIM database (https://www.omim.org/) (accessed on 2 February 2026), and TTD database (http://db.idrblab.net/ttd/) (accessed on 2 February 2026) using “Pancreatic carcinoma” as the keyword. The search results from the three databases were merged and deduplicated to construct the PDAC disease target set. The BPA-related target set and PDAC disease target set were imported into the Venny 2.1 platform (https://bioinfogp.cnb.csic.es/tools/venny/) (accessed on 2 February 2026) to generate a Venn diagram and obtain the intersection targets.

### 4.4. PPI Network Construction and Core Hub Gene Screening

The intersection targets were imported into the STRING database (version 12.0; https://cn.string-db.org/) (accessed on 2 February 2026) to construct a protein–protein interaction (PPI) network. Parameter settings: species was set to “Homo sapiens,” the interaction confidence threshold was set to 0.400 (medium confidence), and disconnected nodes were hidden. The network data generated by STRING were imported into Cytoscape software (version 3.10.3) for visual analysis, and the Maximal Clique Centrality (MCC) algorithm of the CytoHubba plugin (version 0.1) was used to rank the topological importance of network nodes and screen the top 10 core hub genes.

### 4.5. Functional Enrichment Analysis

Gene Ontology (GO) and Kyoto Encyclopedia of Genes and Genomes (KEGG) pathway enrichment analyses were performed on the intersection targets using the DAVID bioinformatics resource (https://david.ncifcrf.gov/) (accessed on 4 February 2026). (https://davidbioinformatics.nih.gov/) (accessed on 4 February 2026). The GO analysis encompassed three categories: Biological Process (BP), Cellular Component (CC), and Molecular Function (MF). The enrichment results were sorted in ascending order by *p* value, and the top 10 significantly enriched terms (*p* < 0.05) were selected and visualized using the SRplot platform (https://www.bioinformatics.com.cn/srplot) (accessed on 4 February 2026).

### 4.6. Molecular Docking

The two-dimensional structure of BPA was downloaded from the PubChem database (https://pubchem.ncbi.nlm.nih.gov/ (accessed on 6 February 2026), CID: 6623, .sdf format), converted to a three-dimensional structure using Chem3D software (version 23.1.1), and then transformed to .pdb format via Open Babel (version 3.1.1). Hydrogen atoms were added to the BPA molecule using AutoDock 4.2.6, Gasteiger charges were assigned, and the molecule was set as a flexible ligand, followed by export as a .pdbqt file.

Protein entries corresponding to the core targets were retrieved from the UniProt database, and crystal structures with X-ray diffraction (X-Ray) as the resolution method and high resolution were selected from the PDB database (https://www.rcsb.org/) (accessed on 6 February 2026). The corresponding .cif files were downloaded from the PDB database, imported into PyMOL (version 3.1.5.1) for removal of water molecules and heteroatoms, and exported as .pdb files. AutoDock 4.2.6 was then used to add polar hydrogen atoms to the protein, which was set as a rigid receptor, and exported as a .pdbqt file.

The .pdbqt files of the ligand and receptor were imported into AutoDock Vina (version 1.1.2). The docking grid box dimensions were set to 60 Å × 60 Å × 60 Å, centered on the protein active pocket. Run parameters were set as follows: cpu = 10, exhaustiveness = 10, num_modes = 20, and energy_range = 3. The binding affinity of BPA with each target was evaluated based on the binding free energy (ΔG), and the conformation with the lowest binding energy was selected for visualization analysis using PyMOL (version 3.1.5.1).

Docking protocol validation and score interpretation. It should be noted that this study follows the conventional analytical paradigm of network toxicology, which focuses on systematic discovery of potential key targets through multi-target screening and binding affinity comparison, rather than precise structural validation of a single complex. Consequently, systematic redocking experiments were not performed. For targets with available co-crystallized ligands in the PDB (e.g., PARP1, PDB: 4R6E; BCL2L1, PDB: 4QVF), the top docking poses were visually confirmed to be located within the known ligand-binding cavities, providing qualitative validation of the grid box placement. The binding ΔG values reported here are in silico predictions generated by AutoDock Vina. They serve as estimates for comparative ranking among targets and binding conformations, and should not be equated with experimentally determined binding affinities. Experimental validation, such as surface plasmon resonance or isothermal titration calorimetry, would be required to confirm the actual binding affinities.

### 4.7. Molecular Dynamics Simulations

The BPA–ESR1 complex was selected for MD simulation not solely based on its marginally lower docking score, but because ESR1 is a well-characterized receptor for environmental estrogens and represents a biologically plausible target for BPA-mediated endocrine disruption (PDB ID: 1L2I). This does not imply that ESR1 is the sole or uniquely dominant target; other high-affinity candidates such as PARP1 and BCL2L1 deserve equal attention in future experimental studies. The simulation was performed using the open-source and versatile GROMACS suite. Specifically, the GROMACS 2024.4 software package was employed to investigate the stability of the protein–ligand complex in an aqueous environment over a duration of 100 ns. PyMOL (version 3.1.5.1) was used to separately extract the protein and small-molecule ligand PDB files from the complex, and OpenBabel GUI (version 2.4.1) was subsequently used to convert the ligand PDB file into MOL2 format. Following preprocessing of the complex structure using SPDBV (version 4.10), the AMBER99SB-ILDN all-atom force field was adopted to describe the protein molecule, while the ligand topology was generated using the Sobtop 1.0 (dev5) (http://sobereva.com/soft/Sobtop) (accessed on 10 March 2026) tool with GAFF2 force field parameters. The system was placed in a dodecahedral periodic boundary condition box with a minimum distance of 1.2 nm between the solute and the box walls. The solvation process employed the TIP3P explicit water model, and system electroneutrality was achieved by adding Na^+^/Cl^−^ counterions. The system was first energy-minimized using the steepest descent algorithm until the maximum force converged below 1000 kJ·mol^−1^·nm^−1^. Subsequently, a two-stage equilibration strategy was adopted: first, the system temperature was stabilized at 300 K under the NVT ensemble using the leap-frog integrator (time step of 2 fs); then, the pressure was adjusted to 1 bar under the NPT ensemble with position restraints through Berendsen pressure coupling (time step of 1 fs, to ensure adequate relaxation of the system during the pressure equilibration phase). Long-range electrostatic interactions were handled using the Particle Mesh Ewald (PME) method to ensure computational accuracy. The final production phase consisted of a 100 ns MD simulation with an integration time step of 1 fs, in which bonds involving hydrogen atoms were constrained using the LINCS algorithm. Trajectory data were collected at a high sampling frequency for subsequent structural visualization and dynamic analysis. This simulation protocol provides a reliable dynamic basis for elucidating the molecular mechanisms underlying protein–ligand interactions. During trajectory visualization using VMD (version 1.9.3), periodic boundary condition (PBC) jumps were observed in the system. This discontinuous trajectory behavior arose from molecules crossing the box boundaries during the simulation, leading to the selection of incorrect periodic images during calculation. The issue was resolved by post-processing the trajectory using the gmx trjconv command with the -pbc nojump and -pbc mol -center -ur compact options. Trajectory analysis plots were generated using DuIvyTools (version 0.6.0) (https://pypi.org/project/DuIvyTools/#files) (accessed on 11 March 2026).

Based on the PC1–PC2 reaction coordinates obtained from principal component analysis (PCA), a two-dimensional free energy landscape (FEL) was constructed using the gmx sham script and visualized using DuIvyTools (version 0.6.0). The color scale was calibrated to Gibbs free energy (0–20 kJ/mol), and global minimum conformations and metastable states were identified through contour topology.

### 4.8. TCGA Clinical Data Validation and Stage-Specific Expression Analysis

It should be emphasized that the TCGA-PAAD dataset does not contain information regarding BPA exposure; therefore, the following expression analysis was de-signed solely to characterize the stage-specific expression trends of the core hub genes in PDAC, and not to infer causality between BPA exposure and gene expression changes.

RNA sequencing data (HTSeq-TPM format) and corresponding clinical information for the PAAD cohort were downloaded from the UALCAN database (https://ualcan.path.uab.edu/) (accessed on 24 April 2026). Inclusion criteria: (1) tumor samples with complete AJCC pathological staging information (Stage I–IV); (2) normal controls consisting of paired adjacent non-tumor tissues. Samples with missing clinical information or inadequate data quality were excluded.

R software (version 4.3.1) was used for data analysis. Based on the PPI network topology analysis results and literature reports, 10 core genes were selected for TCGA validation. Samples were classified into Normal, Stage I, Stage II, Stage III, and Stage IV groups according to the “ajcc_pathologic_tumor_stage” field. The Kruskal–Wallis H test was employed to compare overall differences in gene expression among groups, followed by Dunn’s post hoc test for pairwise comparisons. The ggplot2 package was used to generate box plots displaying the expression distribution of each gene across different clinical stages. The significance level was set at *p* < 0.05.

## 5. Conclusions

Collectively, these findings suggest that ESR1-mediated estrogen signaling disruption may represent a potential molecular event linking BPA exposure to PDAC. Our study provides a computational framework and a set of testable hypotheses for future investigation into the pancreatic toxicity of environmental endocrine-disrupting chemicals. The predicted BPA–target interactions, including those with ESR1, PARP1, and other core hub genes, require experimental validation before their biological relevance can be confirmed.

## Data Availability

The data that support the findings of this study are available from the corresponding author upon reasonable request.

## Supplementary Materials

The following supporting information can be downloaded at: https://www.mdpi.com/article/10.3390/ijms27146439/s1.
